# EDITOR’S MESSAGE / MESSAGE DE LA RÉDACTION

**DOI:** 10.29173/jchla29855

**Published:** 2025-04-01

**Authors:** Jessica McEwan

**Affiliations:** JCHLA/JABSC Editor

Welcome to our spring issue of the JCHLA. I hope the weather is warming wherever you are, bringing with it a sense of hope and possibility.

Our issue opens with an invitation from the co-chairs of the CHLA 2025 conference program committee to join them in Vancouver for this year’s conference. The conference will be organized around the theme of “Building Futures Together”.

In book reviews, M. Caines considers *Managing health sciences libraries in a time of change*, a new offering in the Medical Library Association books series exploring the modern-day responsibilities of those who manage health sciences libraries.

Our second book review, by A. Headrick, considers *Building health sciences library collections: a handbook*. This 2023 publication presents itself as a reference guide for collection managers, exploring current themes as they relate to the health sciences context. Topics include collection diversity and inclusiveness, deselection, open educational resources, non-traditional online collections, as well as aspects specific to medicine, nursing, and allied health.

Happy reading!

**Jessica McEwan**

*JCHLA/JABSC Editor*

*Email:* editor@chla-absc.ca

Bienvenue au numéro de printemps du JABSC. J'espère que le temps se fait plus clément où que vous soyez, apportant avec lui un sentiment d'espoir et de possibilité.

Notre numéro débute par une invitation des coprésidents du comité du programme de la conférence ABSC 2025 à les rejoindre à Vancouver pour la conférence de cette année. La conférence a pour thème « Construire l'avenir ensemble ».

Dans la section critiques de livres, M. Caines examine *Managing health sciences libraries in a time of change*, une nouvelle parution dans la série de livres de la Medical Library Association explorant les responsabilités modernes de ceux qui gèrent les bibliothèques des sciences de la santé.

Notre deuxième critique de livre, rédigée par A. Headrick, porte sur *Building health sciences library collections: a handbook*. Cette publication de 2023 se présente comme un guide de référence pour les gestionnaires de collections, explorant les thèmes actuels dans le contexte des sciences de la santé. Les sujets abordés comprennent la diversité et l'inclusion en ce qui a trait au développement de collection, la désélection, les ressources éducatives ouvertes, les collections en ligne non traditionnelles, ainsi que des thèmes spécifiques à la médecine, aux soins infirmiers et aux professions paramédicales.

Bonne lecture!

**Jessica McEwan**

*Directrice de la rédaction JABSC/JCHLA*

*Courriel:* editor@chla-absc.ca

